# Performance of Whole Blood Stimulation Assays for the Quantification of SARS-CoV-2 Specific T-Cell Response: A Cross-Sectional Study

**DOI:** 10.3390/diagnostics12061509

**Published:** 2022-06-20

**Authors:** Federica Bergami, Francesca Arena, Eleonora Francesca Pattonieri, Marilena Gregorini, Federica Meloni, Massimo Abelli, Elena Ticozzelli, Giorgia Testa, Daniele Lilleri, Irene Cassaniti, Fausto Baldanti

**Affiliations:** 1 Microbiology and Virology Unit, Fondazione IRCCS Policlinico San Matteo, 27100 Pavia, Italy; federica.bergami01@universitadipavia.it (F.B.); francesca.arena01@universitadipavia.it (F.A.); d.lilleri@smatteo.pv.it (D.L.); f.baldanti@smatteo.pv.it (F.B.); 2 Nephrology, Dialysis and Transplantation Unit, Fondazione IRCCS Policlinico San Matteo, 27100 Pavia, Italy; e.pattonieri@smatteo.pv.it (E.F.P.); m.gregorini@smatteo.pv.it (M.G.); 3 Department of Internal Medicine and Medical Therapeutics, University of Pavia, 27100 Pavia, Italy; f.meloni@smatteo.pv.it; 4 General Surgery 4 Unit-Abdominal Transplantation, Fondazione IRCCS Policlinico San Matteo, 27100 Pavia, Italy; m.abelli@smatteo.pv.it (M.A.); e.ticozzelli@smatteo.pv.it (E.T.); 5 Pediatrics Unit, Fondazione IRCCS Policlinico San Matteo, 27100 Pavia, Italy; g.testa@smatteo.pv.it; 6 Department of Clinical, Surgical, Diagnostics and Pediatric Sciences, University of Pavia, 27100 Pavia, Italy

**Keywords:** COVID-19, T-cell response, IGRA

## Abstract

Since the identification of the new severe acute respiratory syndrome virus 2 (SARS-CoV-2), a huge effort in terms of diagnostic strategies has been deployed. To date, serological assays represent a valuable tool for the identification of recovered COVID-19 patients and for the monitoring of immune response elicited by vaccination. However, the role of T-cell response should be better clarified and simple and easy to perform assays should be routinely introduced. The main aim of this study was to compare a home-made assay for whole blood stimulation with a standardized ELISpot assay design in our laboratory for the assessment of spike-specific T-cell response in vaccinated subjects. Even if a good correlation between the assays was reported, a higher percentage of responder subjects was reported for immunocompromised subjects with ELISpot assay (56%) than home-made whole blood stimulation assay (33%). Additionally, three commercial assays were compared with our home-made assay, reporting a good agreement in terms of both positive and negative results.

## 1. Introduction

At the end of December 2019, new severe acute coronavirus syndrome 2 (SARS-CoV-2) was identified in China as the causative agent of coronavirus disease 2019 (COVID-19) that rapidly spread all over the world [1]. In late 2020, anti-SARS-CoV-2 vaccines were introduced, and mRNA BNT162b2 vaccine [2] was the first authorized, showing 95% protection against SARS-CoV-2 infection in a phase II/III trial [3]. Another mRNA-based vaccine, mRNA-1273 [4], showed a similar effect. Besides the antibody level elicited after vaccination, the T-cell memory response induced by the vaccine may have a crucial role in the long-term protection against SARS-CoV-2 infection and disease. 

In natural SARS-CoV-2 infection setting, it has been shown that T cell-mediated immunity (CMI) is responsible for direct interaction with viral infected cells and is involved in the regulation of humoral response [5]. The paramount importance of the CMI and its protective role had been also studied in MERS [6] and SARS infections [7]. In particular, studies on SARS-CoV-1 have shown that T cell memory lasts up to 11 years [8]. It is known that SARS-CoV-2 induces a similar IFN-γ producing Th1 type immune response as other viral infections [9]. To date, persistence of SARS-CoV-2 immune response after natural infection up to 15 months has been demonstrated [10].

Currently, different assays for the quantification of INF-γ after antigenic stimulation (Interferon-gamma release assays, IGRA) have been adapted and developed for monitoring of SARS-CoV-2 T-cell response, including enzyme-linked immune-sorbent analysis (ELISA), histochemical based enzyme-linked immunospot (ELISpot), or flow cytometry [9,11,12,13]. Of these, ELISA and ELISpot do not reveal the cellular source of cytokines, while flow cytometry allow the analysis of cell function and their phenotype in parallel. On the other hand, flow cytometry approach requires highly specialized personnel and it is poorly standardized. 

In the context of SARS-CoV-2 vaccination, there is an urgent need of standardization of methods for the assessment of adaptive T-cell response elicited by vaccination, especially in immunocompromised subjects. The aim of our study is to set-up a simple “home-made” method for the quantification of Spike-specific T-cell response in vaccinated healthy subjects and immunocompromised patients. As gold standard we used an ELISpot assay developed in our institute [10]. Additionally, three commercial assays for the evaluation of Spike-specific produced IFNγ were used.

## 2. Materials and Methods

### 2.1. Study Setting

Samples from 95 healthcare workers (HCW; 60 females and 35 males; median 47.5, range 25–69) and 55 immunocompromised subjects (IC), including hemodialysis patients and solid organ transplant recipients (16 females and 39 males; median 56.5, range 22–74) were analyzed. Whole blood and serum samples were collected six months after BNT162b2 vaccination in both groups. Peripheral blood mononuclear cells (PBMC) were isolated from heparin-treated blood by standard density gradient centrifugation and used for ELISpot assay. Whole blood was used for IFNγ whole blood assay and lymphocyte counts. Serum was used for Spike SARS-CoV-2 IgG serology. The study was approved by the local Ethics Committee (Comitato Etico Area Pavia) and Institutional Review Board (P-20210000232). All the subjects signed informed written consent.

### 2.2. Peptide Pools 

Peptide pools (15mers, overlapping by 10 amino acids, Pepscan, Lelystad, The Netherlands) representative of the Spike protein (S) were used at final concentration of 0.225 µg/mL. Phytoheamagglutinin (PHA; 5 µg/mL) and superantigen staphylococcal enterotoxin B (SEB; 10 µg/mL) were used as positive controls in ELISpot assay and IFN γ whole blood assay, respectively. 

### 2.3. ELISpot Assay 

Ninety-six-well plates (Merck, Darmstadt, Germany) were coated with IFN γ monoclonal capture antibody and stored overnight at 4 °C. After two hours blocking with culture medium, 200,000 cell/well were incubated with stimulating agents: PHA (positive control), and S peptide pool. Medium only was used as negative control. Plates were maintained overnight at 37 °C (5% CO_2_). After multiple wash, anti-IFNγ biotinylated antibody was added and incubated 90 min at 37 °C. Any excess of unbound detection antibodies was removed by washing and streptavidin-alkaline phosphatase conjugate was added and incubated at 37 °C (5% CO_2_). Finally, substrate 5-bromo-4-chloro-3-indolyl phosphatase/nitro blue tetrazolium (BCIP/NBT) was added for 20 min at room temperature. Plates were washed under running water. AID ELISPOT reader system from Autoimmune Diagnostika GmbH (Strasburg, Germany) was used for spots count. 

Results were given as IFNγ spot forming units (SFU)/10^6^ PBMC, after subtracting medium alone response. Valid results were considered when in presence of PHA higher than 100 IFNγ SFU/200,000 cells and medium lower than 5 IFNγ SFU/200,000 cells. Antigen responses higher than 10 IFNγ SFU/10^6^ PBMC were considered to be positive [10].

### 2.4. Home-Made IFNγ Whole Blood Assay

One mL of whole blood was stimulated with the same S peptide pool used for ELISpot assay or SEB as positive control. Unstimulated whole blood was used as negative control. Whole blood was maintained overnight at 37 °C (5% CO_2_). Then, plasma was collected and stored at −80 °C. S-specific IFNγ levels were evaluated using ELISA assay, according to manufacturer’s instructions (Quantikine ELISA, R&D systems, Minneapolis, MN, USA). S-specific IFNγ levels of negative control was subtracted from unstimulated one and normalized on lymphocyte count (BD Lyric flow cytometer, Franklin Lakes, NJ). The detection limit of the test was 0.149 pg/mL. IFNγ levels higher than 10 pg/mL were considered positive. 

Home-made whole blood interferon-gamma release assay (HM-WB IGRA) was then compared with three CE-IVD commercial assay for the quantification of SARS-CoV-2 specific IFNγ production. In detail, 45 samples were tested with Covi-FERON (SD Biosensor, Suwon-si, Republic of Korea) and Quan-T-cell (Euroimmun, Lubeck, Germany) and results were compared with those obtained by HM-WB IGRA. Ten samples were tested with QuantiFERON SARS-CoV-2 (Qiagen, Hilden, Germany) and results were compared with those obtained by HM-WB IGRA.

### 2.5. Statistical Analysis

GraphPad Prism 8.3.0 GraphPad Software, La Jolla, CA, USA) was used for statistical analyses. A two-sided *p* value < 0.05 was considered statistically significant. Data were described with the median and interquartile range (IQR) if continuous and as counts and percentage if categorical. Comparison between two groups was performed using the Mann–Whitney U (unpaired samples) or Wilcoxon (paired samples) test while Spearman’s test was used for the correlation analysis. Fisher’s exact test was used for comparison of categorical variables.

## 3. Results

### 3.1. Correlation between Whole Blood Stimulation and ELISpot Assays in HCWs and ICs

Results obtained by HM-WB IGRA were correlated with those obtained by ELISpot assay in 150 subjects (95 HCWs and 55 ICs). Overall, 116/150 subjects were positive for ELISpot assay; of them, 93 (80.2%) were positive also for HM-WB IGRA. On the other hand, 34/150 subjects were negative for ELISpot assay and, of them, 28 (82.4%) were also negative for HM-WB IGRA (Table 1). 

**Figure 1:**
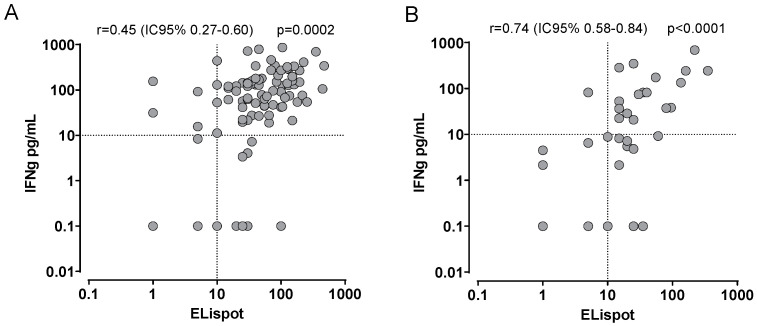
Correlation between HM-WB IGRA (IFNγ pg/mL) and ELISpot assay in HCWs (**A**) and ICs (**B**). Each dot represents a single sample; r and *p* value are given in the graph. HCWs: healthcare workers; ICs: immunocompromised subjects.

Focusing on the performance of the assay in HCWs and ICs, we observed a better correlation between the two assays in ICs than in HCWs (Figure 1A,B). In detail, 30/55 ICs were positive for ELISpot assay and, of them, 19/30 (63.3%) tested positive also for HM-WB IGRA. On the other hand, 25/55 were negative of ELISpot assay and, of them, 24 (96%) were confirmed as negative when tested for HM-WB IGRA. In HCWs cohort, 86/95 subjects were positive for ELISpot assay and, of them, 75/86 (86.2%) were also positive for HM-WB IGRA. Nine subjects showed ELISpot below the cut-off and four of them (44.4%) were also negative for HM-WB IGRA.

### 3.2. Semi-Quantitative Agreement between HM-WB IGRA and ELISpot Assay

**Figure 2:**
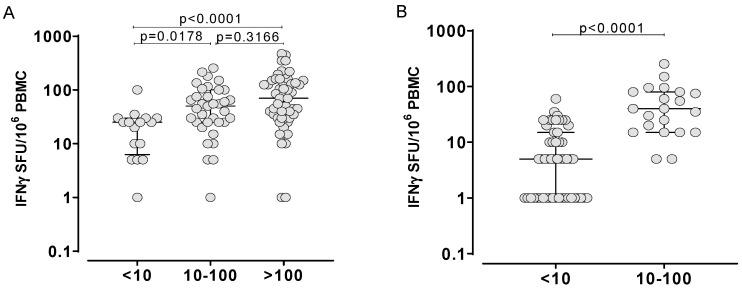
Distribution of SFU/million PBMC based on the IFNγ production measured by HM-WB IGRA is shown in HCWs (**A**) and ICs (**B**). Subjects were divided into three groups based on the level of IFNγ measured by HM-WB IGRA: negative (<10 pg/mL), positive at low/medium level (ranging from 10 to 100 pg/mL) and positive at high level (>100 pg/mL). Median SFU/million PBMC were measured in all the three groups and the p value for each comparison is given.

We analyzed the number of SFU/million PBMC obtained by ELISpot assay in the overall 150 subjects, stratified according to IFNγ production level obtained by HM-WB IGRA. In detail, 52/150 (34.7%) showed a negative IFNγ production level (IFNγ < 10 pg/mL), 47/150 (31.3%) tested positive for IFNγ at medium level (IFNγ level ranging from 10–100 pg/mL) and the remaining 51/150 (34.0%) showed high IFNγ (higher than 100 pg/mL). Looking at HCWs, 16/95 (15.8%) showed a negative IFNγ production level (IFNγ < 10 pg/mL). Thirty-four out of 95 (35.8%) were positive at low/medium level (IFNγ level ranging from 10–100 pg/mL) and the large majority (51/95; 53.7%) showed high levels of IFNγ response. On the other hand, the large majority of ICs showed negative immune response by HM-WB IGRA (39/55; 70.9%) and only 16/55 (29.1%) showed low/medium levels of IFNγ production. None showed IFNγ higher than 100 pg/mL. The median level of SFU/million PBMC for each group of IFNγ response was compared in both HCWs (Figure 2A) and ICs (Figure 2B).

### 3.3. Commercial Assays for the Quantification of IFNγ Production in SARS-CoV-2 Vaccinated Subjects

**Figure 3:**
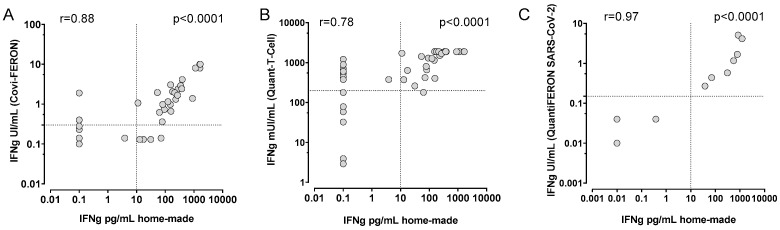
Comparison of home-made WB stimulation assay with Covi-FERON (**A**), Quant-T-Cell (**B**) and QuantiFERON SARS-CoV-2 (**C**) tests. Correlation was determined using Spearman correlation test and r and p value were given for each comparison.

In a subset of samples, comparison between HM-WB IGRA and commercial assays was performed. In detail, Covi-FERON and Quant-T-Cell were tested in parallel in 42 samples and results were compared with those obtained by our HM-WB IGRA assay. Covi-FERON tested positive in 27 samples (25 of them tested positive also for home- HM-WB IGRA) while 16 samples tested negative (12/16 were also negative for HM-WB IGRA) (Figure 3A). Overall, the agreement for positive results was 93% and the agreement for negative results was 75%. On the other hand 36 samples tested positive by Quant-T-Cell assay (28/36 positive for HM-WB IGRA) and 7 were negative by Quant-T-Cell assay (6/7 negative for HM-WB IGRA) (Figure 3B). Overall, the agreement for positive results was 78% and the agreement for negative results 86%. In a small subset of samples, QuantiFERON SARS-CoV-2 was tested in comparison with HM-WB IGRA (Figure 3C), showing a good correlation (r = 0.97).

## 4. Discussion

The understanding of SARS-CoV-2 cell-mediated response in vaccinated frail patients is still a critical issue, in order to define immunization strategies and patient management. In case of vaccinated immunosuppressed subjects, including transplanted patients, monitoring of immune response elicited by vaccination might be important for the identification of those subjects with higher risk of developing disease after infection [13]. At the same time, it has been largely demonstrated that T-cell response against SARS-CoV-2 might be elicited even in absence of detectable humoral response [9,14]. Lastly, in the era of new variants, it has been demonstrated that SARS-CoV-2 T-cell response seems to be less affected by the mutations occurring in SARS-CoV-2 variants [15,16,17].

There is an urgent need to assess simple and standardized methods for the evaluation of T-cell mediated response against SARS-CoV-2. In this study we settled a simple and easy-to-perform assay for the evaluation of S-specific cell-mediated response, by stimulating whole blood and quantifying IFNγ release. The method showed a good correlation with our previously developed *in-house* ELISpot assay, in both healthcare workers and immunocompromised subjects. Overall, 90% and 83% of healthcare workers tested positive for ELISpot assay and HM-WB IGRA, respectively, suggesting the lower sensitivity of the latter one. Similarly, a higher percentage of responder subjects was reported for immunocompromised subjects with ELISpot assay (56%) than respect to HM-WB IGRA (33%). Additionally, three commercial assays were compared with our HM-WB IGRA, reporting a good agreement in terms of both positive and negative results.

As major limitation of the study, only BNT162b2 vaccinated subjects were considered and unexposed donors were not included as control group, thus the correct estimation of the cut-off might be further explored. Additionally, no clinical correlation was provided, thus, a clear association between T-cell response and risk of SARS-CoV-2 infection or disease was not obtained in this study. In the next future, a longitudinal monitoring of T-cell response in vaccinated immunocompromised subjects is needed in order to evidence possible marker of infection risk and infection-related diseases.

## Figures and Tables

**Table 1:** Agreement between ELISpot and HM-WB IGRA in 150 samples.

	ELISpot		
HM-WB IGRA	Positive	Negative	Total
Positive	93	6	99
Negative	23	28	51
Total	116	34	150

Legend: HM-WB IGRA: Home-made whole blood Interferon-gamma release assay; ELISpot: enzyme-linked immunospot assay.

## Data Availability

Data available on request due to restrictions (privacy and ethical).
